# Exploring Older Women’s Attitudes to and Experience of Treatment for Advanced Ovarian Cancer: A Qualitative Phenomenological Study

**DOI:** 10.3390/cancers13061207

**Published:** 2021-03-10

**Authors:** Lucy Dumas, Emma Lidington, Laura Appadu, Philippa Jupp, Olga Husson, Susana Banerjee

**Affiliations:** 1 Gynaecology Unit, Royal Marsden NHS Foundation Trust, London SW3 6JJ, UK; lucy.dumas@rmh.nhs.uk (L.D.); laura.appadu@rmh.nhs.uk (L.A.); philippa.jupp@nhs.net (P.J.); 2 Division of Clinical Studies, Institute of Cancer Research, Sutton SM2 5NG, UK; olga.husson@icr.ac.uk; 3 Clinical Research & Development, Royal Marsden NHS Foundation Trust, London SW3 6JJ, UK; emma.lidington@rmh.nhs.uk; 4 Department of Medical Oncology, Netherlands Cancer Institute—Antoni Van Leeuwenhoek, 1066 CX Amsterdam, The Netherlands

**Keywords:** ovarian cancer, qualitative research, thematic analysis, geriatric, lived experience, treatment preference, chemotherapy

## Abstract

**Simple Summary:**

Older women with ovarian cancer often receive less anti-cancer treatment than younger women despite evidence showing they may benefit from similar levels of treatment. Little is known, however, about older women’s preferences toward chemotherapy and treatment experience. We aimed to understand the lived experience of older women with ovarian cancer undergoing chemotherapy though interviews and focus groups. Participants expressed a strong desire to undergo full treatment to improve survival for themselves and for their families. Women did not see their age as a reason to have less intensive treatment. Despite feeling overwhelmed with information and daily tasks due to fatigue, participants did not want cancer to interfere with their daily lives. Women felt distressed by logistical issues with transportation and communication between healthcare providers; however, they still felt positive about their care experience and desire for treatment. Older women may benefit from additional help to support effective communication around treatment preferences.

**Abstract:**

Older women with ovarian cancer more often receive less intensive treatment and early discontinuation compared to younger women. There is little understanding of older women’s treatment experience and whether this contributes to declining intensive treatment. We aimed to explore the lived experience of older patients with advanced ovarian cancer undergoing chemotherapy, their treatment preferences and treatment burden. We conducted a phenomenological qualitative study with 15 women who had completed at least three cycles of first-line chemotherapy for advanced epithelial ovarian cancer, aged 65 years or older at the first cycle, at one tertiary cancer centre. We conducted interviews and focus groups and analysed the transcripts using inductive thematic analysis. Women reported a strong preference for active treatment despite treatment burden and toxicities. Participants undertook treatment to lengthen their lives for themselves and their families. Participants did not see age as a barrier to treatment. Patients expressed determination not to let cancer interfere with daily life. Women felt overwhelmed with information and struggled with daily tasks due to fatigue. Logistical issues, such as transportation and ineffective communication between healthcare providers, caused substantial distress. Despite these logistical burdens and toxicities, participants were positive about their care experience and desire for anticancer treatment. Older women may benefit from additional support to facilitate effective communication during the early stages of treatment.

## 1. Introduction

Research has established that older women with ovarian cancer have disproportionately poorer survival outcomes than younger women [1]. Older women receive less intensive treatment which may in part may explain the discrepancy [2,3,4,5]. 

Some of this variation is likely due to clinicians appropriately not pursuing intensive treatment with older women at higher risk of treatment toxicity [6]. However, without utilising an objective geriatric assessment, as recently recommended in American Society of Clinical Oncology guidance [7], older patients’ fitness may be underestimated or modifiable risk factors may not be addressed leading to unnecessary under-treatment. In these cases, psychological and practical factors, including inherent age bias, may contribute to the provision of less intensive care [8]. The Elderly Women with Ovarian Cancer (EWOC-1) study demonstrated that in women over age 70, standard three-weekly carboplatin with paclitaxel chemotherapy was superior to single-agent three-weekly carboplatin without significant compromise on tolerability. The findings strongly support offering combination chemotherapy in newly diagnosed older women as in younger patients [9]. 

From the patient perspective, it is unclear if treatment burden encourages older women to decline intensive treatment, further contributing to treatment variation. Previous research has found there is no difference between older and younger women’s desire for cure from gynaecological cancers and older patients often choose to undertake additional treatment for survival benefits [10,11]. While research has found that older adults tend to place more value on quality of life than younger cancer patients, older patients still place importance on survival [12,13,14]. 

To help better understand the treatment preferences and treatment experiences of older women with advanced ovarian cancer undergoing chemotherapy, we explored these topics in a qualitative phenomenological study.

## 2. Materials and Methods

Patients currently receiving chemotherapy for advanced epithelial ovarian cancer or in follow-up at the Royal Marsden NHS Foundation Trust were identified in clinic lists by the treating team between April and July 2019. Eligible women were aged 65 years or older at the time of their first chemotherapy cycle, had completed at least three cycles in the first line setting or at relapse, and were proficient in English. This age threshold was chosen to align with the American Society of Clinical Oncology guidance on the management of older patients being considered for systemic anti-cancer therapy and the European Medical Association cut-off from a pharmacovigilance perspective [7,15]. Patients with significant cognitive impairment, mental health problems or severely unwell, as determined by the clinician, were ineligible. Care was taken to invite patients that were unwell but potentially fit enough to participate where possible. Recruitment occurred concurrently with data analysis. Participants were purposively sampled for breadth in disease and toxicity severity. Potential participants were posted patient information sheets followed-up by telephone after one week.

Participants chose to take part in focus groups or individual interviews facilitated by two of the authors (EL or LD). The authors chose to use both methods of data collection for data completeness. Interviews allowed the researchers to explore personal experiences in-depth, while focus groups elicited shared and discordant opinions and beliefs [16]. Individual interviews also enabled women who were too unwell to attend the focus group to take part in the study, a key group of interest in this study. Participants provided written informed consent and completed a short background questionnaire before taking part. Focus groups took place in hospital meeting rooms. Facilitators followed a semi-structured interview schedule which was reviewed by patients and members of the public (Table 1). Discussions were audio-recorded. Field notes were taken during focus groups. 

The Royal Marsden Committee for Clinical Research reviewed and approved the study in October 2018 (SE764). The data that support the findings of this study are available from the corresponding author upon reasonable request.

Analysis followed the six phases of inductive thematic analysis described by Moules: (1) engaging with the data, (2) generating initial codes, (3) identifying themes, (4) reviewing themes, (5) defining and naming themes and (6) writing the report [17].

Audio recordings were transcribed verbatim. LD and EL conducted initial coding, following Saldana’s techniques [18]. To reduce bias, each researcher undertook initial coding independently before reviewing and agreeing final initial codes. Researchers consulted field notes while generating initial codes and during reconciliation to justify code choice. 

The agreed initial codes and related excerpts were then manually analysed by LD and EL to identify potential themes and subthemes. Again, field notes added further insight to analytical decisions. The study team reviewed potential themes and subthemes to assess coherence and relevance. LD conducted two further telephone interviews at this stage. LD and EL independently coded the new transcripts and checked the codes against existing data. As no new codes were identified, the study team felt confident in theoretical saturation and data collection ended [19]. Themes and subthemes were then reviewed by the whole study team to refine the names and definitions.

## 3. Results

**Figure 1:**
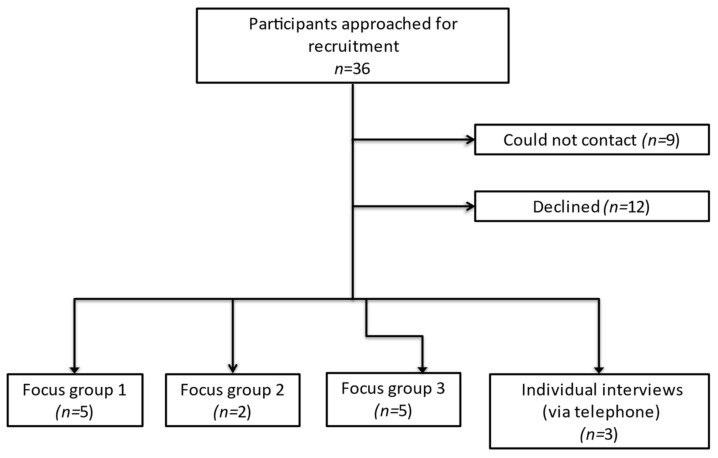
Flowchart of participant recruitment.

Of 36 eligible patients invited to the study, 15 agreed to take part (Figure 1). Reasons for declining the study included time or logistical constraints, burden of hospital appointments and feeling too unwell. Focus groups each took two hours whilst interviews ranged from 29 to 60 min.

Table 2 summarises the participant characteristics. Mean age at diagnosis was 75.0 years (range 68–89 years). Mean age at participation was 78.4 years (range 71–90 years). Twelve (80%) participants received standard three-weekly carboplatin and paclitaxel chemotherapy as first-line treatment and nine (60%) received treatment for relapsed disease. Three (20%) patients received single-agent carboplatin. In total, 4 (27%) patients required a dose-reduction during chemotherapy due to toxicity. In total, 4 (27%) had caring responsibilities for a family member. Six of the 15 patients were receiving chemotherapy at the time of study entry (all for recurrent disease). The remaining nine were in follow up, six following first-line treatment and three following treatment for recurrent disease.

Initial codes were incorporated into three themes: Multifactorial decision-making, burden of logistical issues and coping with side effects.

### 3.1. Theme: Multifactorial Decision-Making

#### 3.1.1. Subtheme: Reception and Retention of Information Clouds Decisions

Participants frequently felt overwhelmed by the volume of information shared at diagnosis and memories around diagnosis were often unclear. Shock at the diagnosis, particularly for patients who had enjoyed relative health throughout their lives, was apparent and contributed to feeling overwhelmed.

*“When you’re first diagnosed, it’s such a shock to you, you don’t absorb everything that’s being said.”*

*(Patient 4, 82 years at diagnosis, receiving first-line chemotherapy)*

*“I sort of went into zombie mode, it never occurred to me to say no.”*

*(Patient 12, 70 years at diagnosis, in follow up from first-line chemotherapy)*

Fatigue and severe illness further inhibited understanding of complex information and involvement in treatment decisions. Participants appreciated pamphlets received during their first appointments, but many admitted not reading them.

*“I was desperately tired at this stage and an awful lot of information is given to you”.*

*(Patient 14, 72 years at diagnosis, in follow up from first-line chemotherapy)*

Participants felt decisions, including clinical trial participation, were rushed due to pressure to start treatment. For most, this was a stressor, however, one woman felt it reduced the worry around the decision.

*“Well I had to make the decision on the spot…so there was no time to think about it.”*

*(Patient 14, 72 years at diagnosis, in follow up from first-line chemotherapy)*

For some participants, the internet was a crucial source of information, challenging the notion that older patients are less adept with technology. In contrast, some participants felt strongly that information found online, particularly around prognosis, would be unhelpful and cause distress.

*“I can’t bear looking on the internet, I just don’t want to know.”*

*(Patient 8, 69 years at diagnosis, receiving chemotherapy for relapsed disease)*

This avoidance was more frequently expressed by patients who had relapsed disease. Others wanted more information, with uncertainty around prognosis a clear source of anxiety.

*“You’ve gone through six months of chemo feeling blimmin awful for no … you know, nobody knows the answer.”*

*(Patient 2, 78 years at diagnosis, receiving chemotherapy for relapsed disease)*

#### 3.1.2. Subtheme: Lengthening Life Expectancy

An important, common expression was the clear determination to be treated with no reference to age or comorbidities. A sense of deciding between life and death was apparent and participants worried that age might be considered to be a reason against treatment.

*“I was told I could go profoundly deaf … but I had to take that chance.”*

*(Patient 7, 74 years at diagnosis, in follow up from first-line chemotherapy)*

*“I would have done anything to have treatment, gone through anything.”*

*(Patient 1, 77 years at diagnosis, in follow up from first-line chemotherapy)*

Many participants feared treatment reduction or discontinuation. One patient suffering from severe peripheral neuropathy with significant functional limitation admitted needing persuasion to allow chemotherapy dose-reduction, fearing this would shorten her life-expectancy. Negative impacts on functional ability and quality of life did not appear to deter women from treatment.

*“I’d still want to be alive because there’s other things I’m sure I’d be able to do.”*

*(Patient 1, 77 years at diagnosis, in follow up from first-line chemotherapy)*

One participant, who had not received full standard chemotherapy and cytoreductive surgery owing to anaesthetic risk, expressed disappointment. All participants, when asked individually, agreed they would undertake treatment again given their experience with treatment and side-effects so far.

#### 3.1.3. Subtheme: Family Influence

Participants commonly reported that they underwent treatment for their family members, rather than themselves. Women often expressed concerns about the ramifications of their death on their family. For some participants, adult children played an active role in treatment decisions.

*“They didn’t want me to have six months or less to live, it’s not nice for them knowing one’s going to die so that’s why they said yes to chemo, to see how much longer it would give them.”*

*(Patient 2, 78 years at diagnosis, receiving chemotherapy for relapsed disease)*

Participants caring for dependent spouses faced additional pressure during treatment.

*“I think the thing that put most pressure on me was my husband being ill.”*

*(Patient 13, 76 years at diagnosis, in follow up after first-line chemotherapy)*

Patients with dependent spouses also expressed concern over how their spouse would manage without them. Worry that their spouse would become a burden to their children with their death compelled some women to undertake treatment.

*“I want to be here for him, rather than leaving him for my family to look after”*

*(Patient 2, 78years at diagnosis, receiving chemotherapy for relapsed disease)*

### 3.2. Theme: Burden of Logistical Issues

#### 3.2.1. Subtheme: Care Coordination

Generally, participants felt they had excellent care and support from the cancer centre and expressed sincere gratitude.

*“It’s like a blanket around you isn’t it.” *

*(Patient 1, 77 years at diagnosis, in follow up from first-line chemotherapy)*

However, women were concerned about the lack of communication between their oncology team and other specialists at external institutions, particularly those with co-morbidities. Some patients described waiting months to be seen by another specialist after referral. 

Many participants found navigating logistical issues difficult, forming a substantial discussion in each focus group. Patients felt burdened by the responsibility of communicating with hospital staff to manage appointments. This was particularly difficult in the early stages of treatment when concurrently dealing with severe physical illness. Participants had varied access to informal charitable support, largely due to geographical barriers.

*“It’s just these things on the ground that you have to do as a patient when you’re feeling exhausted.” *

*(Patient 14, 72 years at diagnosis, in follow up after first-line chemotherapy)*

Participants also discussed a lack of communication between primary care and the cancer centre and often did not realise primary care could be involved in cancer treatment or support. Women felt involvement may be limited by resource strain and a lack of continuity in provider. Some participants had experienced a significant delay before diagnosis culminating in emergency presentation. This understandably had an impact on willingness to engage with primary care throughout treatment.

*“I don’t actually know who my doctor is.” *

*(Patient 4, 82 years at diagnosis, receiving first-line chemotherapy)*

#### 3.2.2. Subtheme: Transport

Difficulties arranging transport to appointments caused significant stress. Many patients were unaware of hospital transport until after attending a number of appointments. Information received about transport frequently came from administrative staff rather than the clinical team, leading to delays in acquiring assistance. The need to arrive earlier or leave later than appointments for those using hospital transport added to the stress and fatigue associated with hospital visits.

#### 3.2.3. Subtheme: Informal Support

Friends and family played an invaluable role in supporting women with the burden of treatment by attending clinic or chemotherapy appointments or providing emotional support.

*“Oh your family back you up don’t they.” *

*(Patient 2, 78 years at diagnosis, receiving chemotherapy for relapsed disease)*

Faith was brought up independently by a number of participants. Women found solace in faith during treatment, potentially as a way to cope with the lack of control of treatment outcomes. For some, the regular routine of worship and associated community support provided comfort during treatment.

*“The only thing I can do is put my hand in the hand of god and you, I can’t do anything more”*

*(Patient 8, 69 years at diagnosis, receiving chemotherapy for relapsed disease)*

### 3.3. Theme: Side-Effects

#### 3.3.1. Subtheme: Weighed Down by Side-Effects

Chemotherapy tolerability varied widely. Some women expressed surprise at how few side-effects they experienced. For many, however, issues such as myalgia and arthralgia were difficult to tolerate. Peripheral neuropathy and its impact on function, particularly for participants who previously enjoyed physical activity, was a significant and long-term issue.

Overwhelmingly, the most profound issue reported was fatigue, or as subjects termed it, “utter exhaustion”. Participants described fatigue as the most challenging aspect of their treatment experience, able to manage only the most basic activities of daily living.

*“I was incredibly weak and then you still have to do things and you can’t manage it.”*

*(Patient 11, 70 years at diagnosis, in follow up after first-line chemotherapy)*

*“It’s a matter of dragging my body around to keep up with essentials.”*

*(Patient 14, 72 years at diagnosis, in follow up from first-line chemotherapy)*

For some, this level of fatigue continued for months after treatment. Severe fatigue had ramifications beyond physical limitations. Many participants expressed a real sense of loss as a result of their cancer treatment. Participation in previously enjoyed activities, even simple endeavours such as walking the dog, were “taken away”. Fatigue and weakness, which for some persisted for months after treatment, contributed to a loss of confidence in their ability to remain independent or travel alone. Those who lived alone also reported a fear of falling or having an accident and being unable to access help. Some participants felt this had an effect on mood, admitting they “get very depressed at times”.

#### 3.3.2. Subtheme: Determination Not to Let Cancer Interfere

Despite the clear physical impact of the treatment, participants had a shared ambition not to let the diagnosis or treatment impact on day-to-day life, maintaining independence and self-sufficiency. One participant, discharged from hospital following a midline-laparotomy, found online videos for instructions on how to safely sit up in bed. Others refused aids from well-meaning family members to avoid being seen as ill. Participants expressed a reluctance to approach their medical team with what they perceived as minor symptoms to avoid being a ‘burden’. 

Women prioritised participating in leisure and hobby activities. Those who were previously physically active viewed their current treatment as a temporary setback. An unwillingness to be hampered by the medical diagnosis was clear.

*“I just carry on as normal, I do my Pilates, I go to a club, but I’ve forgotten what it’s like to feel normal.”*

*(Patient 1, 77 years at diagnosis, in follow up from first-line chemotherapy)*

Despite physical limitations, in particular fatigue and weakness, participants continued with life to the best of their abilities with a markedly stoic outlook.

*“I live life normally and I will go on like that until it’s my time to go.”*

*(Patient 11, 70 years at diagnosis, in follow up after first-line chemotherapy)*

## 4. Discussion

Despite most participants receiving standard treatment, women overwhelmingly preferred treatment with the chance of lengthening survival despite side-effects and would undertake treatment again. Participants chose to undergo treatment for their own sake or to please family members. Though patients struggled with fatigue and logistical challenges, these older women were determined to maintain independence and continue treatment.

Our findings suggest that the preferences of older patients with ovarian cancer toward anti-cancer treatment are unlikely to substantially contribute towards lower treatment intensity. Whilst the decision not to offer cytoreductive surgery or platinum-doublet chemotherapy may be based on sound medical concerns, this study demonstrates that older women do not consider age a barrier to treatment. All participants expressed the preference for full standard treatment, including those who suffer treatment-related toxicities or were not offered standard treatment. 

Recent developments provide strong evidence that geriatric assessment can not only improve the prediction of treatment tolerance and mortality [7], but also, when combined with targeted interventions, reduce the frequency of severe treatment-related toxicity [20,21]. For example, fatigue, highlighted by participants as one of the most debilitating issues, may contribute to the need to reduce chemotherapy dose-intensity. However, fatigues is often multifactorial and addressing factors such as anaemia, vitamin deficiencies, thyroid dysfunction, physical exercise, nutrition and social support could reduce fatigue and allow patients to maintain treatment intensity, meeting both patient needs and preferences [22]. 

Treatment preferences and expected outcomes should be clearly discussed with all patients irrespective of age. As demonstrated here, older women may be more unwell and less able to participate in treatment decisions after likely emergency presentation or delayed diagnosis [23,24]. Reduced information reception and retention also limited involvement in treatment decisions. A systematic review found that audio-recording consultations improved information retention, particularly in older adults, and question lists encouraged active participation [25]. With the use of the Internet demonstrated by older women in this study, these tools, coupled with online resources, could be useful adjuncts to standard consultations. Older women may also benefit from opportunities to re-discuss treatment aims later in the pathway when patients are less overwhelmed. 

Diversifying methods for information provision may help address the aversion to prognostic information some women described. Avoidant coping styles have been shown to be maladaptive and associated with distress and low emotional wellbeing [26]. However, evidence on coping strategies in older adults have shown mixed results, with one study showing older adults are more likely to adopt avoidant strategies and another finding they are more likely to exhibit passive reactions [27,28]. Further research is needed to explore how best to support older women in coping with a cancer diagnosis.

As many older adults face social isolation and functional limitations cancer treatment may impose a large burden [29]. Logistical issues coordinating and attending appointments with multiple providers posed a particular challenge in this study. While this did not hamper willingness to undertake treatment, efforts should be taken to assess the burden of treatment from the patient perspective, particularly for older patients with multi-morbidity [30]. Practical issues, such as transport, need to be incorporated into routine assessment. A number of methods previously shown to lessen the burden of treatment were employed by participants, such as enlisting the help of others and turning to faith to cope [31]. Ascertaining levels of social support is essential for older patients who may live alone.

These findings may not be applicable to other hospitals as this study was conducted in a single cancer centre. The small sample size inherent in qualitative research also limits generalisability. One focus group contained only two participants due to last minute cancellations for the reasons described. This limited the ability to explore similar and discordant beliefs in one of the groups but further serves to highlight the changing health status and logistical burden for these patients. This study is also subject to survivor bias as only patients who received at least three cycles of systemic therapy were included, meaning patients whose condition deteriorated early on or who chose not to undertake treatment were excluded. 

We also acknowledge the possibility of selection bias. While investigators made every effort to include patients with a range of disease severities, it was considered unethical to invite severely unwell patients, causing undue stress. This likely introduced some level of bias. Additionally, despite the use of both focus groups and individual interviews, patients declined due to illness and logistical constraints, potentially further introducing bias. Even with these limitations, the interviews describe important experiences of cancer management in older women with ovarian cancer from the patient perspective. 

Future work should build on the current study and recent research to ensure older women with advanced ovarian cancer receive optimal treatment. A longitudinal survey study looking at treatment preferences and health-related quality of life would mitigate survivor bias and improve generalisability.

## 5. Conclusions

The older women in this study were overwhelmingly positive about their experience of cancer care and desire for anticancer treatment, despite facing treatment burden and therapy-related toxicities. Older women may face additional challenges in terms of information retention and managing medical comorbidities. Additional methods of delivering information could be useful to improve patient centred decision making.

## Figures and Tables

**Table 1:** Semi-structured interview schedule.

Question
1. What were the biggest challenges you faced while receiving treatment?
2. Do you feel, before starting treatment, that you had a good idea of the potential risks and benefits of treatment?
3. Is there any information you wish you had received that you didn’t prior to starting chemotherapy?
4. What were your main goals of treatment/Why did you decide to have treatment?
5. How much did the opinions of your clinicians impact on your decision to have treatment?
6. How much did the feelings and opinions of your family and friends impact on your decision to have treatment?
7. Knowing now the side-effects you have experienced, would you make the same decision again to undergo treatment?
8. When you think about your health, what would you say the term quality of life means to you?
9. With that in mind, in what ways has your diagnosis and treatment for ovarian cancer affected your quality of life?
10. If you experienced side effects during treatment, did you feel well-supported?
11. How well do you think your GP * and other community teams were kept informed of your progress during treatment?

* general practitioner.

**Table 2:** Patient characteristics.

Patient Characteristic	*n*	(%)
Living situation		
Lives alone	6	(40.0)
Lives with spouse	6	(40.0)
Lives with other family members	2	(13.3)
Lives in sheltered accommodation	1	(6.7)
Has caring responsibilities	4	(26.7)
Employment status		
Retired	14	(93.3)
Financial impact		
Cancer has had no financial impact	13	(86.7)
Cancer has had a little financial impact	2	(13.3)
Stage at diagnosis		
1	0	(0)
2	4	(26.7)
3	9	(60.0)
4	2	(13.3)
Primary treatment		
Platinum doublet chemotherapy	12	(80.0)
Single-agent carboplatin	3	(20.0)
Surgery	11	(73.3)
Primary treatment tolerance		
No delays	6	(40.0)
Delay 1 week or less	3	(20.0)
Delay >/= 2 weeks	2	(13.3)
Dose reduction at beginning	0	(0)
Dose reduction during chemotherapy	4	(26.7)
Has had disease recurrence	8	(53.3)
Second line treatment		
Chemotherapy (doublet)	3	(20.0)
Chemotherapy (single agent)	2	(13.3)
Clinical Trial	2	(13.3)

## Data Availability

The data that support the findings of this study are available from the corresponding author upon reasonable request.

## References

[1] Cabasag C.J., Butler J., Arnold M., Rutherford M., Bardot A., Ferlay J., Morgan E., Møller B., Gavin A., Norell C.H. (2020). Exploring variations in ovarian cancer survival by age and stage (ICBP SurvMark-2): A population-based study. Gynecol. Oncol..

[2] Dumas L., Bowen R., Butler J., Banerjee S. (2021). Under treatment of older patients with newly diagnosed epithelial ovarian cancer remains an issue. Cancers.

[3] Ferrero A., Fuso L., Tripodi E., Tana R., Daniele A., Zanfagnin V., Perotto S., Gadducci A. (2017). Ovarian cancer in elderly patients: Patterns of care and treatment outcomes according to age and modified frailty index. Int. J. Gynecol. Cancer.

[4] Fourcadier E., Trétarre B., Gras-Aygon C., Ecarnot F., Daurès J.P., Bessaoud F. (2015). Under-treatment of elderly patients with ovarian cancer: A population based study. BMC Cancer.

[5] Tew W.P., Muss H.B., Kimmick G.G., Von Gruenigen V.E., Lichtman S.M. (2014). Breast and ovarian cancer in the older woman. J. Clin. Oncol..

[6] Tew W.P., Fleming G.F. (2015). Treatment of ovarian cancer in the older woman. Gynecol. Oncol..

[7] Mohile S.G., Dale W., Somerfield M.R., Schonberg M.A., Boyd C.M., Burhenn P.S., Canin B., Cohen H.J., Holmes H.M., Hopkins J.O. (2018). Practical assessment and management of vulnerabilities in older patients receiving chemotherapy: ASCO guideline for geriatric oncology. J. Clin. Oncol..

[8] Woodard S., Nadella P.C., Kotur L., Wilson J., Burak W.E., Shapiro C.L. (2003). Older women with breast carcinoma are less likely to receive adjuvant chemotherapy: Evidence of possible age bias?. Cancer.

[9] Falandry C., Savoye A.M., Stefani L., Tinquaut F., Lorusso D., Herrstedt J., Bourbouloux E., Floquet A., Brachet P.E., Zannetti A. (2019). EWOC-1: A randomized trial to evaluate the feasibility of three different first-line chemotherapy regimens for vulnerable elderly women with ovarian cancer (OC): A GCIG-ENGOT-GINECO study. J. Clin. Oncol..

[10] Nordin A.J., Chinn D.J., Moloney I., Naik R., de Barros Lopes A., Monaghan J.M. (2001). Do elderly cancer patients care about cure? Attitudes to radical gynecologic oncology surgery in the elderly. Gynecol. Oncol..

[11] Sattar S., Alibhai S.M.H., Fitch M., Krzyzanowska M., Leighl N., Puts M.T.E. (2018). Chemotherapy and radiation treatment decision-making experiences of older adults with cancer: A qualitative study. J. Geriatr. Oncol..

[12] Meropol N.J., Egleston B.L., Buzaglo J.S., Benson A.B., Cegala D.J., Diefenbach M.A., Fleisher L., Miller S.M., Sulmasy D.P., Weinfurt K.P. (2008). Cancer patient preferences for quality and length of life. Cancer.

[13] De Celis E.S.P., Li D., Sun C.-L., Kim H., Twardowski P., Fakih M. (2018). Patient-defined goals and preferences among older adults with cancer starting chemotherapy (CT). J. Clin. Oncol..

[14] Shrestha A., Martin C., Burton M., Walters S., Collins K., Wyld L. (2019). Quality of life versus length of life considerations in cancer patients: A systematic literature review. Psychooncology.

[15] Committee for Human Medicinal Products (2007). Adequacy of Guidance on the Elderly Regarding Medicinal Products for Human Use.

[16] Lambert S.D., Loiselle C.G. (2008). Combining individual interviews and focus groups to enhance data richness. J. Adv. Nurs..

[17] Nowell L.S., Norris J.M., White D.E., Moules N.J. (2017). Thematic Analysis: Striving to Meet the Trustworthiness Criteria. Int. J. Qual. Methods.

[18] Saldaña J. (2015). The Coding Manual for Qualitative Researchers.

[19] Saunders B., Sim J., Kingstone T., Baker S., Waterfield J., Bartlam B., Burroughs H., Jinks C. (2018). Saturation in qualitative research: Exploring its conceptualization and operationalization. Qual. Quant..

[20] Li D., Sun C.L., Kim H., Chung V., Koczywas M., Fakih M., Chao J., Chien L., Charles K., Fernandes Dos Santos Hughes S. (2020). Geriatric assessment-driven intervention (GAIN) on chemotherapy toxicity in older adults with cancer: A randomized controlled trial. J. Clin. Oncol..

[21] Mohile S.G., Mohamed M.R., Culakova E., Xu H., Loh K.P., Magnuson A., Flannery M.A., Ramsdale E.E., Dunne R.F., Gilmore N. (2020). A geriatric assessment (GA) intervention to reduce treatment toxicity in older patients with advanced cancer: A University of Rochester Cancer Center NCI community oncology research program cluster randomized clinical trial (CRCT). J. Clin. Oncol..

[22] Fabi A., Bhargava R., Fatigoni S., Guglielmo M., Horneber M., Roila F., Weis J., Jordan K., Ripamonti C.I. (2020). Cancer-related fatigue: ESMO Clinical Practice Guidelines for diagnosis and treatment. Ann. Oncol..

[23] Tate A.R., Nicholson A., Cassell J.A. (2010). Are GPs under-investigating older patients presenting with symptoms of ovarian cancer Observational study using General Practice Research Database. Br. J. Cancer.

[24] National Cancer Intelligence Network Routes to Diagnosis 2015 Update: Ovarian Cancer n.d. http://www.ncin.org.uk/publications/data_briefings/.

[25] Gaston C.M., Mitchell G. (2005). Information giving and decision-making in patients with advanced cancer: A systematic review. Soc. Sci. Med..

[26] Costanzo E.S., Lutgendorf S.K., Rothrock N.E., Anderson B. (2006). Coping and quality of life among women extensively treated for gynecologic cancer. Psychooncology.

[27] Baitar A., Buntinx F., De Burghgraeve T., Deckx L., Schrijvers D., Wildiers H., van den Akker M. (2018). The influence of coping strategies on subsequent wellbeing in older patients with cancer: A comparison with 2 control groups. Psychooncology.

[28] Hernández R., Calderon C., Carmona-Bayonas A., Rodríguez Capote A., Jara C., Padilla Álvarez A., Gómez-Camacho M.D.L.N., Beato C., Castelo B., Majem M. (2019). Differences in coping strategies among young adults and the elderly with cancer. Psychogeriatrics.

[29] Long Roche K., Angarita A.M., Cristello A., Lippitt M., Haider A.H., Bowie J.V., Fader A.N., Tergas A.I. (2016). “Little big things”: A qualitative study of ovarian cancer survivors and their experiences with the health care system. J. Oncol. Pract..

[30] Leppin A., Montori V., Gionfriddo M. (2015). Minimally Disruptive Medicine: A Pragmatically Comprehensive Model for Delivering Care to Patients with Multiple Chronic Conditions. Healthcare.

[31] Ridgeway J.L., Egginton J.S., Tiedje K., Linzer M., Boehm D., Poplau S., de Oliveira D.R., Odell L., Montori V.M., Eton D.T. (2014). Factors that lessen the burden of treatment in complex patients with chronic conditions: A qualitative study. Patient Prefer Adherence.

